# Development of a mHealth Intervention (TechCare) for First Episode Psychosis: A Focus Group Study With Mental Health Professionals

**DOI:** 10.1192/bjo.2022.195

**Published:** 2022-06-20

**Authors:** Nadeem Gire, Naeem Mohmed, Mick McKeown, Joy Duxbuy, Nusrat Husain

**Affiliations:** 1 The University of Central Lancashire, Preston, United Kingdom; 2 Lancashire & South Cumbria NHS Foundation Trust, Preston, United Kingdom.; 3 Manchester Metropolitan University, Manchester, United Kingdom; 4 The University of Manchester, Manchester, United Kingdom

## Abstract

**Aims:**

Research in the area of mHealth, has shown much promise in the development of mobile phone interventions which look at the assessment and treatment in real-time of psychiatric disorders. Within the context of Severe Mental Illnesses (SMI), such as psychosis, communication and understanding between health professionals and service users in the reporting of distress and reoccurrence of symptoms is essential in reducing the chances of relapse. An alternative pathway which uses mobile technology to engage with services, may hold the key to gaining a deeper understanding of the lived experiences of those with mental health difficulties, in particular experiences of recovery from SMI's. AIM: The aim of the study was to explore the perspectives and opinions of health professionals on the development and refinement of the TechCare App for psychosis. A qualitative approach was adopted for data collection, which provided an understanding of factors in relation to the development of the intervention.

**Methods:**

A total of two focus groups were held with health professionals to elicit their views on optimising the utility of the TechCare App. The total sample size for the focus groups was n = 16 with a total of 6 males and 10 females. This qualitative study was part of a feasibility study, investigating a novel intervention (TechCare) (Husain et al., 2016; Gire et al., 2021) which monitored participants symptoms and provided a tailored psychosocial response in real-time. Data obtained from the focus groups was transcribed. Framework analysis were used to analyse the data for emerging themes, focusing on feasibility, acceptability and further development.

**Results:**

The key themes that emerged from the data were; access and usage of digital technologies, implications for clinical practice, challenges & barriers to delivery and development and refinement considerations for the TechCare App.

**Conclusion:**

Results of the focus group with health professionals provided a unique perspective of conducting mHealth research within an EIS context, and the differing challenges professionals anticipated facing in delivering the TechCare App intervention. The main finding of the focus group was that professionals saw the potential for the TechCare App to increase access to digital technologies, providing service users with an alternative medium to communicate with EIS health professionals. However, the participants felt that despite mHealth Apps being a useful platform to deliver interventions, face-to-face contact should remain an important aspect of routine care.

